# Malaysian Nurses' Knowledge of Radiation Protection: A Cross-Sectional Study

**DOI:** 10.1155/2021/5566654

**Published:** 2021-08-04

**Authors:** Aisyah Mohd Rahimi, Intan Nurdin, Shahrina Ismail, Azira Khalil

**Affiliations:** Faculty of Science and Technology, Universiti Sains Islam Malaysia, 71800 Nilai, Negeri Sembilan, Malaysia

## Abstract

Radiology is a vital diagnostic tool for multiple disorders that plays an essential role in the healthcare sector. Nurses are majorly involved in a healthcare setting by accompanying patients during the examination. Thus, nurses tend to be exposed during inward X-ray examination, requiring them to keep up with radiation use safety. However, nurses' competence in radiation is still a concept that has not been well studied in Malaysia. The study aimed to define the level of usage understanding and radiation protection among Malaysian nurses. In this research, a cross-sectional survey was conducted among 395 nurses working in hospitals, clinics, and other healthcare sectors in Malaysia. The survey is based on the developed Healthcare Professional Knowledge of Radiation Protection (HPKRP) scale, distributed via the online Google Forms. SPSS version 25.0 (IBM Corporation) was used to analyze the data in this study. Malaysian nurses reported the highest knowledge level in radiation protection with a mean of 6.03 ± 2.59. The second highest is safe ionizing radiation guidelines with 5.83 ± 2.77, but low knowledge levels in radiation physics and radiation usage principle (4.69 ± 2.49). Therefore, healthcare facilities should strengthen the training standards for all nurses working with or exposed to radiation.

## 1. Introduction

The radiological examination using X-rays remains the most widely used ionizing radiation in the medical field. It is liable for the most significant human-made radiation exposure to the worldwide population [1]. Based on the United Nations Scientific Committee on the Effects of Atomic Radiation (UNSCEAR) study, approximately 4 billion X-ray examinations are conducted every year [2]. X-ray examinations are primary imaging tools for diagnosing tests that have been used for decades. They allow us to see what is in our body without performing an incision [3]. This X-ray procedure is performed quickly and painlessly. X-ray imaging is the most important imaging used in clinical settings to help doctors diagnose and monitor certain body conditions and medical health [4] in most hospitals and clinics worldwide. The invention of X-rays was a major development in medicine. X-ray imaging tests are a valuable diagnostic instrument for various procedures and examinations [5].

Ionizing radiation has been strictly controlled in that it could pose a harmful effect on human health. To protect both humans and the environment from the adverse effects of ionizing radiation, radiation safety standards have been developed [6]. Nurses can engage in a variety of medical ionizing radiation procedures. For example, when X-ray imaging involves exposing patients, it sometimes needs to be conducted in wards for patients who cannot walk to the X-ray room, exposing them to scattered radiation [7]. Deterministic effects are also referred to as tissue reactions, depending on the amounts of radiation exposure. At higher doses, health effects such as skin injury and hair loss may be more severe [7–9]. This endangers the safety of nurses and patients when exceeding the radiation threshold [10]. Besides, tumors and leukemia are the stochastic effects of radiation, arising throughout extended periods caused by genetic material changes [8, 9]. Therefore, the possible stochastic risks of ionizing radiation exposure should be known by nurses, while patients should be wary of both deterministic and stochastic risks.

The previous statement shows that nurses play a vital role in the healthcare sector since they accompany patients most of the time. Thus, during the inward radiation, they can be exposed to radiation while working behind protective barriers. There are two probable sources of radiation exposure from the occupational viewpoint [11]. The true radiation source is the X-ray tube, but workers may be inadvertently exposed to the primary beam in rare situations. The second source of radiation exposure is the patient. The interaction between the primary X-ray beam and the area of the patient's body getting imaged creates scattered radiation originating from the patient in all directions. However, in most situations, occupational exposure's key determinant is nurses' proximity to the patient as exposures are taking place. According to the WHO, IAEA, and ICRP, radiation protection should be standardized to the highest possible safety level [12, 13]. The lack of ionizing radiation awareness among nurses can cause them to be unable to effectively protect themselves and their patients [14–16].

Nurses' knowledge of the ALARA (as low as reasonably achievable) concept of radiation safety is important. ALARA is a principle of protection intended to reduce radiation doses and radioactive material releases. ALARA focuses on legal dosage limits for regulatory compliance, serving as a standard for all radiation safety programs rather than just best practice [17]. It is possible to conform to ALARA principles by knowing and using the main parameters, shielding, time, and distance. Usually, patient dosage reduction also reduces the dose to nurses [18]. In radiological practice, the design of optimized procedures is also an important part of radiation protection [19]. Hence, nurses should know radiation use and protection to ensure their safety involving ionizing radiation. However, the competence of nurses in radiation remains a poorly studied term. The previous study shows that there has been a general lack of radiation education among nurses [7, 20].

To ensure that nurses possess adequate radiation understanding, described as the person's ability to use radiation safely, it is crucial to identify their specific deficiencies and assess the current level of understanding. From the previous study conducted in Finland, nurses' radiation understanding has been assessed using the Healthcare Professional Knowledge of Radiation Safety (HPKRP) scale to assist these efforts. This scale includes an indication of three radiation knowledge areas [6, 21]. The first is radiation physics and the radiation use principle in the medical setting. In contrast, the second area is radiation protection, which protects patients and nurses from harmful ionizing radiation. Finally, there are guidelines for using medical radiation relating to national and international guidelines for safe ionizing radiation. In Malaysia, the study that addresses this issue was conducted in 2014 but not using the HPKRP scale, which encourages the need to perform a new current study [22]. Therefore, this study was conducted to investigate a cross-sectional study of Malaysian nurses' knowledge of radiation use and radiation safety using HPKRP scale items. The HPKRP scale is chosen in this research because it was developed from the previous research [6] to be used in educational, clinical practice, and research settings.

This study aimed to characterize the understanding of radiation usage and protection by Malaysian nurses. In this study, target groups were nurses who use ionizing radiation to serve in hospitals, clinics, and other healthcare sectors around Malaysia. Malaysian nurses working in the first-aid clinics, operating theatres, medical wards, and radiology departments were invited to answer the questionnaire. This study's objectives were to investigate Malaysian nurses' current radiation understanding level and identify their understanding of radiation use and protection affected by background factors.

## 2. Methods

### 2.1. Study Design

A cross-sectional questionnaire survey was conducted in which data from nurses working in random hospitals, clinics, and other healthcare sectors across Malaysia were collected simultaneously. The questionnaire was created using a Google Form divided into two sections, with 39 questions in total.

### 2.2. Participant and Settings

In 2019, around 108 thousand (107,830) registered nurses in Malaysia increased compared to the previous year [23]. Based on the sample size calculator, the sufficient data to generalize this research outcome on Malaysia's whole nurse population are *N* = 383 (with 95% confidence interval and 5% margin error) [24]. Therefore, random Malaysian nurses (*N* = 395) serving in first-aid clinics, operating theatres, radiology departments, and medical wards were invited to participate in the research. In Malaysia, nurses exposed to radiation every day typically work in first-aid clinics, operating theatres, and radiology departments. Inclusion requirements for nurses must have been employed in a unit where ionizing radiation can be used or exposed. Nurses received the invitations and questionnaires from the beginning of semester 1 to the middle of semester 1 in 2020.

### 2.3. Instrument

The self-administered questionnaire consisted of two sections. Section one consists of the background question (participant background), while section two is regarding HPKRP scale's items, further classified into another three parts. Demographic (background) questions comprise gender, age, educational level, work experience, and details about the nurses' work unit. On the contrary, the HPKRP scale helped to determine the radiation understanding of involved nurses [6]. The following radiation understanding dimensions or aspects are addressed by the three sections of scope given by (1) radiation physics and radiation use principle that contain 12 items; (2) radiation protection, which has 13 items; and (3) guidelines for safe ionizing radiation use that include 8 items. Each item is evaluated on a Likert scale from 1 to 10, where 1 denotes “no knowledge” and 10 denotes “full knowledge.” The scale reflected the knowledge and understanding of the participants. The HPKRP scale was then designed to collect highly detailed information on participants' self-reported understanding and use of different radiation aspects [21]. It has been psychometrically checked and shown to have much validity of the face, content, and construction [14]. Note that Cronbach's alpha coefficients of the scale ranged from 0.93 to 0.96 with an S-CVI value of 0.83 [21]. This showed that the scale is valid to be used in the research setting.

### 2.4. Data Collection

Random Malaysian nurses working in first-aid clinics, operating theatres, medical wards, and radiology departments were invited to participate in the study, where 395 of them accepted the invitation. All data were collected using the Google Form, in which participants could easily access using a link throughout the distributed message. The participating nurses' response was anonymous and processed according to the published standards of good research practice.

### 2.5. Ethical Consideration

All participants were required to sign an informed consent form before responding to the questionnaire, which explains the introduction of this study, what it will entail, the benefits, risks and discomforts, confidentiality, payment and rewards, and the duration. The authors also stated that responses from the participants would be documented for further study. The data will be safely stored, where only researchers would have access to the data. The data will be deleted after the data analysis is finished and the final paper is accepted for publication. All participants decided to participate in the research, and none declined to answer the questionnaire.

### 2.6. Data Analysis

The data received from the Google Form were evaluated using SPSS version 25.0 (IBM Corporation). Demographic data were evaluated with descriptive statistics and summarized as percentages and frequency, while the data of radiation knowledge were summarized as standard deviation and mean. Moreover, a binary logistic regression and cross-tabulation were employed to analyze whether the radiation knowledge was related to any demographic variables. The outcome variables were then classified from Likert scale responses into dichotomous variables. The first variable is “0” for scores between 1.00 and 4.99, while the second variable is “1” for scores between 5.00 and 10.00, designated “lower” and “higher” knowledge, respectively [21]. The results were investigated and presented as distribution percentage and probability, where statistical significance was set at *p* < 0.05.

## 3. Results

Most nurses were female (360, 91.1%) aged between 18 and 27 years (155, 39.2%). The nurses had various educational backgrounds: 332 (84.1%) had diploma level education, 60 (15.2%) had a bachelor's degree, and 3 (0.8%) had a master's degree. Most nurses received medical radiation education (269, 68.1%), while only 126 out of 395 nurses did not receive medical radiation education. Most participating nurses are from first-aid clinic units, with 186 nurses (47.1%) (see Table 1).

For the dimension of radiation physics and radiation use principles (Table 2), the lowest reported knowledge is on stochastic effects of a certain radiation dose (mean: 4.07; SD: 2.84). The nurses' highest reported knowledge in this section is about the harmful effects of medical radiation (mean: 6.79; SD: 2.63). Next, for radiation protection (Table 2), the highest reported knowledge is about other personnel safety while working in a controlled area that involves radiation exposure (mean: 7.39; SD: 2.57). In contrast, the lowest reported knowledge is on the meaning of the inverse square law in radiation protection (mean: 4.41; SD: 3.13). In the third section of the HPKRP scale, which is guidelines for safe ionizing radiation use, most nurses recorded higher knowledge about the meaning of warning signs regarding radiation safety (mean: 6.99; SD: 2.77). Knowledge about dose limitation in radiation protection was the lowest reported knowledge of participating nurses (mean: 5.07; SD: 3.15).

The correlation of demographic data and radiation knowledge is shown in Tables 3–5. In this research, statistical significance was set at *p* < 0.05, implying a significant correlation between those demographic data and radiation knowledge area. The binary logistic regression and cross-tabulation analysis show that age, nurse's work unit, nurse's working experience, and medical radiation education correlate with each radiation knowledge area. Nurses aged between 28 and 37 years recorded significantly higher levels of radiation physics and radiation use principles than nurses aged 18 to 27 years (*n* = 39, % = 9.9, and *p*=0.03) (see Table 3). Also, nurses who have work experience between 10 and 14 years reported significantly higher levels of this knowledge than nurses with working experience between 0 and 4 years (*n* = 29, % = 7.3, and *p*=0.04). Besides, nurses who work in the radiology department have significantly higher radiation physics levels and radiation use principles than nurses who work in operating theatres (*n* = 19, % = 4.8, and *p*=0.01). In addition, nurses who receive medical radiation education recorded significantly higher levels of this knowledge than nurses who had not received the education (*n* = 167, % = 42.3, and *p* < 0.01).

The correlation between the background data of nurses and radiation protection knowledge area can be seen in Table 4. Nurses who have completed medical radiation education recorded significantly higher levels of this knowledge area than nurses who had not completed the education (*n* = 258, % = 65.3, and *p* < 0.01). The results also show that nurses' age and medical radiation education correlate with guidelines of safe ionizing radiation use (Table 5). Nurses between 28 and 37 years old scored significantly higher in this knowledge than nurses between 18 and 27 years old (*n* = 57, % = 14.4, and *p* < 0.01). Again, nurses who received medical radiation education recorded significantly higher levels of this radiation knowledge area than nurses who had not received the education (*n* = 240, % = 60.8, and *p* < 0.01).

## 4. Discussion

The study found that participating nurses scored higher in their radiation safety expertise than their knowledge of the other two aspects of radiation knowledge (guidelines of radiation use and radiation physics). Here, the average value on the 10-point Likert scale is 6.03. The fact that nurses have such a relatively clear knowledge of radiation safety policies is encouraging, given the importance of radiation protection to those exposed to it [25]. They indicated that staff safety is crucial when operating in a regulated area involving exposure to radiation but lack of understanding of the concept of the inverse square law of radiation protection. The participants rated their knowledge of safety radiation guidelines second best, with a mean Likert score of 5.83 (Table 2). They mentioned being adept at the use of warning signs in radiation protection. Unfortunately, they have less understanding of the concept of dose limitation in radiation safety. Deficiencies in the knowledge of radiation safety precautions and actions have been reported [15], and they should specifically be discussed to guarantee the safe use of radiation [26].

The radiation physics dimension and radiation usage principles were the lowest recorded information levels with a mean Likert scale of just 4.69 (Table 2). The data show that most participating nurses are experts about the harmful effects of medical radiation but not well educated with the ALARA concept and stochastic effects of a certain radiation dose in medical radiation. The lack of knowledge of this main theory (and issues raised earlier) has been noted globally [16, 26]. Another significant outcome is that, as previously stated, nurses felt that they had not undergone adequate radiation education [16]. From the result above, it is clear that medical radiation education is significantly linked with all three areas of radiation knowledge. The need to enhance education is reinforced by further observation of a clear gap in understanding radiation safety between nurses who had not undergone radiation education. The lack of radiation education will put both nurses' and patients' health at high risk [18]. Hence, education is important to ensure that nurses successfully follow the applicable rules and procedures for the safe use of radiation [27], develop a culture of protection among users of radiation, and adhere to national and international standards.

## 5. Conclusion

The study shows that Malaysian nurses are well educated about radiation protection and radiation usage but lack radiation physics knowledge. This has serious implications for both patients and nurses. Training and learning by providing skills for all nurses involved in the ionizing radiation is a key for optimizing quality and education. Therefore, healthcare organizations play an important role in ensuring that all nurses in Malaysia are well educated about radiation knowledge by enhancing education requirements for all nurses who act around or are exposed to radiation. In conclusion, the findings demonstrate the value of radiation knowledge and radiation understanding and, most significantly, the function and role of education to ensure the safe use of medical radiation.

## Figures and Tables

**Table 1:** Demographic details of participating nurses (*N* = 395).

Background variables	*n*	%
Age (years)		
18–27	155	39.2
28–37	104	26.3
38–47	88	22.3
48–57	48	12.2

Gender		
Male	35	8.9
Female	360	91.1

Work experience in years		
0–4	151	38.2
5–9	56	14.2
10–14	57	14.4
15–20	52	13.2
Over 20	79	20.0

Education		
Diploma	332	84.1
Bachelor's degree	60	15.2
Master's degree	3	0.8

Working unit		
Operating theatre	70	17.7
Radiology department	20	5.1
First-aid clinic	186	47.1
Medical ward	119	30.1

Medical radiation education in hours		
No	126	31.9
Yes	269	68.1

**Table 2:** Items of HPKRP scale competence (*N* = 395).

HPKRP scale main factors and items	Mean ± standard deviation
*Radiation physics and radiation use principles*	4.69 ± 2.49
I know how ionizing radiation is produced	4.41 ± 2.76
I know the differences between ionizing and nonionizing radiation	4.20 ± 2.77
I know the differences between electromagnetic and ionizing radiation	4.40 ± 2.90
I know the characteristics and physical features of X-rays	4.96 ± 2.92
I know how the harmful effects of medical radiation are caused	6.79 ± 2.63
I can describe the deterministic effects of certain radiation doses	4.29 ± 2.85
I can describe the stochastic effects of a certain radiation dose	4.07 ± 2.84
I know the justification principles for medical radiation examinations	4.57 ± 2.90
I understand the equations and measures in medical radiation examinations	4.13 ± 2.82
I understand the meaning of the as low as reasonably achievable principle in radiation examinations	4.21 ± 2.98
I know the fundamental principles of radiation protection	5.89 ± 2.92
I have obtained enough education about the use of radiation in medical examinations	4.35 ± 2.72
*Radiation protection and safety*	6.03 ± 2.59
I know how to properly use personal radiation protection equipment (PPE)	7.29 ± 2.62
I know how to properly use the radiation protection equipment for patients	6.65 ± 2.85
I pay attention to the other personnel while working in a controlled area and using radiation	7.39 ± 2.57
I know how to document all the essential information concerning the use of radiation	5.52 ± 3.15
I am aware that information concerning a patient's radiation dose must be written down in patient records	5.44 ± 3.22
I know the protocols concerning radiation workers who are pregnant	6.85 ± 2.90
I try to promote agreed safety protocols concerning radiation dose and radiation usage in my daily work and actions	6.12 ± 3.03
I understand the factors affecting a patient's radiation dose	5.67 ± 3.09
I understand the meaning of the inverse square law in radiation protection	4.41 ± 3.13
I know how to account for differences between adult and child/adolescent patients in radiological examinations	5.27 ± 3.14
I know how to assess my actions critically and comprehensively while working with medical radiation	5.33 ± 3.03
I am aware of the radiation safety arrangements at my work	6.32 ± 2.92
I understand the meaning of radiation safety culture	6.14 ± 2.96
*Guidelines of safe ionizing radiation use*	5.83 ± 2.77
I know the meaning of warning signs regarding radiation safety	6.99 ± 2.77
I observe and notice the warning signs concerning radiation while working in the control area	6.97 ± 2.73
I know how radiation workers' health monitoring has been organized	6.08 ± 3.13
I am aware of the classification of radiation workers	5.47 ± 3.12
I understand the procedures for how radiation exposure in radiation workers is monitored	5.66 ± 3.11
I know how to report abnormal events in radiation usage	5.22 ± 3.15
I understand the situations in which the “abnormal event notification” must be performed	5.22 ± 3.17
I understand the principle of dose limitation in radiation protection	5.07 ± 3.15

This HPKRP scale is credited to T. Schroderus-Salo et al., “Development and validation of a psychometric scale for assessing healthcare professionals' knowledge in radiation protection.”

**Table 3:** Background factors correlated with radiation physics and radiation use principles' knowledge area (*N* = 395).

Independent variable	Outcome variable				
	Lower knowledge		Higher knowledge		Probability (*p*)
	*n*	%	*n*	%	
Age (years)					
18–27 (ref.)					
28–37	65	16.5	39	9.9	0.03
38–47	61	15.4	27	6.8	0.14
48–57	37	9.4	11	2.8	0.06

Gender					
Male (ref.)					
Female	209	52.9	151	38.2	0.95

Work experience in years					
0–4 (ref.)					
5–9	40	10.1	16	4.1	0.58
10–14	28	7.1	29	7.3	0.04
15–20	37	9.4	15	3.8	0.33
Over 20	60	15.2	19	4.8	0.22

Education					
Diploma (ref.)					
Bachelor's degree	30	7.6	30	7.6	0.09
Master's degree	1	0.3	2	0.5	0.29

Working unit					
Operating theatre (ref.)					
Radiology department	1	0.3	19	4.8	0.01
First-aid clinic	127	32.2	59	14.9	0.52
Medical ward	55	13.9	64	16.2	0.08

Medical radiation education					
No (ref.)					
Yes	102	25.8	167	42.3	<0.01

**Table 4:** Background factors correlated with radiation protection and safety knowledge area (*N* = 395).

Independent variable	Outcome variable				
	Lower knowledge		Higher knowledge		Probability (*p*)
	*n*	%	*n*	%	
Age (years)					
18–27 (ref.)					
28–37	40	10.1	64	16.2	0.75
38–47	44	11.1	44	11.1	0.51
48–57	22	5.6	26	6.6	0.44

Gender					
Male (ref.)					
Female	125	31.6	235	59.5	0.94

Work experience in years					
0–4 (ref.)					
5–9	25	6.3	31	7.8	0.71
10–14	17	4.3	40	10.1	0.75
15–20	25	6.3	27	6.8	0.69
Over 20	42	10.6	37	9.4	0.44

Education					
Diploma (ref.)					
Bachelor's degree	20	5.1	40	10.1	0.04
Master's degree	0	0.0	3	0.8	0.99

Working unit					
Operating theatre (ref.)					
Radiology department	1	0.3	19	4.8	0.46
First-aid clinic	79	20.0	107	27.1	0.49
Medical ward	35	8.9	84	21.3	0.21

Medical radiation education					
No (ref.)					
Yes	11	2.8	258	65.3	<0.01

**Table 5:** Background factors correlated with guidelines of safe ionizing radiation use knowledge area (*N* = 395).

Independent variable	Outcome variable				
	Lower knowledge		Higher knowledge		Probability (*p*)
	*n*	%	*n*	%	
Age (years)					
18–27 (ref.)					
28–37	47	11.9	57	14.4	<0.01
38–47	52	13.2	36	9.1	0.04
48–57	26	6.6	22	5.6	0.03

Gender					
Male (ref.)					
Female	142	35.9	218	55.2	0.78

Work experience in years					
0–4 (ref.)					
5–9	27	6.8	29	7.3	0.20
10–14	21	5.3	36	9.1	0.13
15–20	33	8.4	19	4.8	0.53
Over 20	45	11.4	34	8.6	0.11

Education					
Diploma (ref.)					
Bachelor's degree	23	5.8	37	9.4	0.26
Master's degree	1	0.3	2	0.5	0.49

Working unit					
Operating theatre (ref.)					
Radiology department	1	0.3	19	4.8	0.26
First-aid clinic	89	22.5	97	24.6	0.27
Medical ward	38	9.6	81	20.5	0.60

Medical radiation education					
No (ref.)					
Yes	29	7.3	240	60.8	<0.01

## Data Availability

The data used to support the findings of this study are available from the corresponding author upon request (azira@usim.edu.my).
